# Morphological and molecular diversity of *Sclerotinia sclerotiorum* infecting Indian mustard

**DOI:** 10.1007/s42360-018-0054-7

**Published:** 2018-09-17

**Authors:** A. S. Rathi, Minakshi Jattan, Rakesh Punia, Subaran Singh, Pawan Kumar, Ram Avtar

**Affiliations:** 1 Department of Plant Pathology, CCS Haryana Agricultural University, Hisar, Haryana 125 004 India; 2 Department of Genetics and Plant Breeding, CCS Haryana Agricultural University, Hisar, Haryana 125 004 India

**Keywords:** *Sclerotinia sclerotiorum*, Sclerotia, Mycelial, Cultural, Variability

## Abstract

Fourteen isolates of *Sclerotinia sclerotiorum* were collected from different locations of mustard growing regions of India and were studied for cultural, morphological and molecular variability at CCS HAU, Hisar. Variability was observed for colony colour, type of growth, diameter of mycelial growth, sclerotia initiation, number and pattern of sclerotia formation among the isolates. Mycelial growth and sclerotia initiation were faster in Bhiwani isolate as compared to others. Bhiwani isolate was found to be the most diverse and had least similarity with Chhanibari isolate on the basis of molecular variability. Hence, morphological and cultural variability observed in the present investigation is by and large strongly correlated to molecular marker based variability.

## Introduction

*Sclerotinia sclerotiorum* (Lib.) de Bary, is one of the most important and devastating soil inhabiting necrotropic and non-host specific fungal plant pathogens with broad ecological distribution. The fungus infects more than 500 cultivated and wild plant species (Sharma et al. 2015) and causes substantial damage to its host under favourable environments. In India, the disease was of minor importance few decades ago, but in recent years it has become a serious problem in major mustard growing areas (Lodha et al. 1992; Krishnia et al. 2000; Ghasolia et al. 2004). The disease has also been observed to cause heavy losses in yield in Indian mustard (*Brassica juncea*) as the incidence of this disease was noticed up to 72% at some of the locations in Rajasthan (Shivpuri et al. 2000; Ghasolia et al. 2004) and up to 80% in some of the areas in Punjab and Haryana (Kang and Chahal 2000).

This ascomycete fungus causes infection by myceliogenic and carpogenic germination of sclerotia surviving in soil. Once the pathogen is established, it is difficult to manage due to its soil borne nature and wide host range. Control of this disease in mustard is not feasible and economical because with the exception many a time this disease appears at pod formation stage to maturity. The use of resistant varieties is one of the important alternatives to overcome this problem. However, complete resistance to *S. sclerotiorum* is absent in all cultivated rapeseed-mustard crops, though partial resistance was identified in some of the *B. napus* and to a lesser extent *B. juncea* genotypes from China, Australia (Li et al. 2008) and India (Singh et al. 2008). Lack of effective field resistance to Sclerotinia rot in cultivated species of rapeseed-mustard has stimulated the interest of researchers towards finding out variations occurring at the cultural, morphological and molecular characteristics among the isolates from different geographical regions to analyze the changes evolving in the population of *S. sclerotiorum*. Morphological characteristics of *S. sclerotiorum* collected from various hosts have already been reported in literature (Morrall et al. 1972; Willetts and Wong 1980; Ziman et al. 1998; Basha and Chatterjee 2007). Several molecular methods such as amplified fragment length polymorphism (Cubeta et al. 1997), random amplified fragment length polymorphism (Yli-Mattila et al. 2010; Thilagavathi et al. 2013), micro satellite marker (Meinhardt et al. 2002), sequence-related amplified polymorphism (SRAP) technique (Li et al. 2009) and Universal Rice Primer Polymerase Chain Reaction (URP-PCR) (Aggarwal et al. 2008) were used to determine genetic diversity of fungus. However, most of these studies on variability among the isolates of *S. sclerotiorum* were carried out by collecting different isolates from various hosts (Basha and Chatterjee 2007; Goswami et al. 2008; Aldrich-Wolfe et al. 2015; Kapatia et al. 2016). In all living organisms variability is required for their adaptation, survival, development and reproduction depending upon whether sexually or asexually reproducing nature. Necrotrophic pathogens having wide host range may have many diverse genes for virulence or, more likely, because their genes of virulence somehow have much less plant specificity than those of the commonly more specialized pathogens (Agrios 2005). In India, the pathogen was considered to be of more myceliogenic in nature, but now for the last few years carpogenic nature of infection through air borne ascospores has become common that indicate variability in pathogen through hybridization. Undoubtedly, studies have been initiated for finding out the solution to control this disease through genetic resistance by biotechnological methods. Hence, there is a need to find out the diversity analysis of *S. sclerotiorum* infecting Indian mustard as the variation in pathogen affect the success of breeding programme and chemical control strategy. Therefore, the present study was conducted to ascertain the cultural, morphological and molecular variability among different isolates of *S. sclerotiorum* obtained from infected Indian mustard from major mustard growing regions of India.

## Materials and methods

### Collection, isolation, purification and multiplication of *S. sclerotiorum* isolates

Fourteen isolates of *S. sclerotiorum* in the form of sclerotia were collected at the time of harvesting from different mustard growing states viz., Haryana, Punjab and Rajasthan (Table 1) and stored under laboratory conditions in the Oilseeds Section, Department of Genetics and Plant Breeding, CCS Haryana Agricultural University, Hisar, Haryana. These sclerotia were plated on potato dextrose agar (PDA) medium after surface sterilization with 0.1% mercuric chloride solution and incubated at 21 ± 1 °C for 3 days (Kumar et al. 2016). Each isolate was purified by transferring the single hyphal tip on to the fresh medium and prepared the pure culture of each isolate which were further multiplied.

**Table 1:** Isolates of *Sclerotinia sclerotiorum* obtained as sclerotia from infected Indian mustard plants from different mustard growing regions of India

Sr. no.	Name of isolates	Area of collection	Geographical location^a^
1	BWL	Bawal, Haryana	28°.08′N–76°.58′E
2	BWN	Bhiwani, Haryana	28°.47′N–76°.08′E
3	DBW	Dabwali, Haryana	29°.95′N–75°.73′E
4	FTB	Fatehabad, Haryana	29°.31′N–75°.27′E
5	HSR	Hisar, Haryana	29°.15′N–75°.70′E
6	MHG	Mahendragarh, Haryana	28°.28′N–76°.15′E
7	RHK	Rohtak, Haryana	28°.40′N–76°.13′EE
8	SRS	Sirsa, Haryana	29°.53′N–75°.00′E
9	LDH	Ludhiana, Punjab	30°.91′N–75°.85′EE
10	CHBR	Chhanibari, Rajasthan	29°.11′N–75°.20′E
11	HNM	Hanumangarh, Rajasthan	29°.35′N–74°.19′E
12	NHR	Nohar, Rajasthan	29°.11′N–74°.46′E
13	RSN	Raisingh Nagar, Rajasthan	29°.32′N–73°.26′E
14	SGN	Sriganganagar, Rajasthan	29°.92′N–73°.88′E

^a^Source—http://www.worldatlas.com/aatlas/findlatlong.htm

### Cultural and morphological variability

Mycelial disc of 5 mm diameter of each isolate was taken from actively growing colony of 4 days old culture and was transferred on to fresh PDA in Petri plate (90 mm diameter). All the cultures were incubated at 21 ± 1 °C in BOD incubator and observations on the cultural characters viz., colony colour, and type of growth after 96 h and size of mycelial growth (mm) in diameter were recorded at 24, 48, 72 and 96 h after incubations. Four replications with three Petri plates per replication were used for each isolate. The morphological methods as suggested by Morrall et al. (1972) were used for the sclerotia formation i.e., initiation of sclerotia formation in days after incubation (DAI), number of sclerotia formation in plates and pattern of sclerotia formation on PDA in Petri plates.

### Molecular variability

The mycelium of each isolate was grown in potato dextrose broth by incubating at 21 ± 1 °C and 120 rpm. After 5–6 days, mycelium of each isolate was filtered through Whatman filter no. 1, washed twice with the TE buffer, blot dried completely and stored at − 70 °C till DNA isolation. For DNA extraction, the cetyltrimethyl ammonium bromide (CTAB) method of Murray and Thompson (1980) was used with slight modifications. The quantity and quality of DNA samples were tested by submerged horizontal agarose gel (0.8%) electrophoresis (Sambrook et al. 1989) along with a standard marker. The appropriate dilutions of DNA samples were done for the PCR amplifications with Universal Rice Primers (URPs) (Table 2). PCR amplifications were carried out in 10 µl reaction mixture containing 20 ng genomic DNA, 1.5 units of Taq DNA polymerase, 0.2 mM of dNTPs, 1.5 mM MgCl_2_ and 0.2 µM of primer. The optimized PCR program was as follows: an initial step of 5 min at 94 °C, followed by 35 cycles of 1 min at 94 °C, 60 °C annealing temperature for 1 min, 1 min at 72 °C, and a final extension step of 10 min at 72 °C. PCR products were separated on 2.0% agarose gel electrophoresis for better resolution. The DNA ladder (50 bp) was also loaded in the gels to estimate the proper band size of amplified products and photographed using Vilber Lourmat gel documentation system.

**Table 2:** List of Universal Rice Primers (URPs) used in the study

Sr. no.	Primer	Sequence (5′–3′)	Primer orientation
1	URP 1F	ATC CAA GGT CCG AGA CAA CC	F + R
2	URP 2F	GTG TGC GAT CAG TTG CTG GG	F + R
3	URP 2R	CCC AGC AAC TGA TCG CAC AC	F + R
4	URP 4R	AGG ACT CGA TAA CAG GCT CC	F + R
5	URP 6R	GGC AAG CTG GTG GGA GGT AC	F + R
6	URP 9F	ATG TGT GCG ATC AGT TGC TG	F + R
7	URP 13R	TAC ATC GCA AGT GAC ACA GG	F + R
8	URP 17R	AAT GTG GGC AAG CTG GTG GT	F + R
9	URP 25F	GAT GTG TTC TTG GAG CCT GT	F + R
10	URP 30F	GGA CAA GAA GAG GAT GTG GA	F + R
11	URP 32F	TAC ACG TCT CGA TCT ACA GG	F + R
12	URP 38F	AAG AGG CAT TCT ACC ACC AC	F + R

### Statistical analysis

A binary matrix was compiled using numerical system of multivariate analysis. The dendogram was constructed by the unweighted paired group method of arithmetic average (UPGMA) based on Jaccard’s similarity coefficient (Jaccard 1908) with SHAN program of NTSYS-PC.

## Results and discussion

### Cultural and morphological variability

All the fourteen isolates of *S. sclerotiorum* were found be variable to some extent in colony colour, type of growth on the basis of cultural characteristics of mycelium (Table 3). Dabwali (DBW), Fatehabad (FTB), Sirsa (SRS), Chhanibari (CHBR), Nohar (NHR) and Raisingh Nagar (RSN) isolates showed dirty white colony colour, while rest of the isolates showed whitish colony colour particularly Hisar (HSR) isolate. However, Contrary to the present observation, Kumar et al. (2016) reported that HSR isolate had dirty white colony colour indicating the presence of two types at the same location for colony colour. Slight variations can occur for colony colour within isolates collected from different hosts as Sharma et al. (2013) also found differences in colony colour among the isolates as whitish and dirty white, however, off white and grey white colony colour as observed by them were not found in any of the isolates in the present study. However, Ziman et al. (1998) observed a slight variation in colony colour of *S. sclerotiorum* isolates collected from different hosts, which differentiate from white to brown but the white colour was predominant in most of the isolates.

**Table 3:** Cultural and morphological variability among fourteen *Sclerotinia sclerotiorum* isolates collected from Indian mustard

Isolates	Cultural variability						Morphological variability		
	Colony colour	Type of growth	Mycelial growth (diameter in mm)				Sclerotia formation		
			24 h	48 h	72 h	96 h	Initiation (DAI)	Av. no. sclerotia/plate	Pattern
BWL	Whitish	Fluffy and regular	18.3	47.8	74.7	90	5	26	Attached to rim
BWN	Whitish	Sparse and regular	20.7	65.0	90.0	90	4	43	Attached to rim
DBW	Dirty white	Sparse and regular	22.0	62.7	79.0	90	5	54	Double ring near to rim and centre
FTB	Dirty white	Fluffy and regular	7.0	27.7	34.0	90	6	18	Attached to rim
HSR	Whitish	Sparse and regular	15.3	32.3	59.0	90	6	40	Near to rim
MHG	Whitish	Fluffy and regular	7.0	31.3	53.7	90	7	23	Attached to rim
RHK	Whitish	Sparse and regular	11.7	25.0	41.3	90	7	39	Scattered all around
SRS	Dirty white	Fluffy and irregular	3.3	16.3	34.0	90	7	42	Attached to rim
LDH	Whitish	Sparse and regular	3.7	17.7	51.7	90	6	38	Attached to rim
CHBR	Dirty white	Sparse and irregular	7.0	21.2	47.7	90	8	29	Attached to rim
HNM	Whitish	Sparse and regular	29	62.0	86.7	90	4	34	Attached to rim
NHR	Dirty white	Fluffy and irregular	4.3	19.7	41.3	90	6	20	Scattered all around
RSN	Dirty white	Sparse and regular	5.0	15.3	41.3	90	6	28	Attached to rim
SGN	Whitish	Sparse and regular	5.7	12.3	44.0	90	5	30	Near to rim

The variations in type of growth were also observed among the isolates as BWL, FTB and MHG isolates showed fluffy and regular type of growth. However, SRS, and NHR isolates showed fluffy but irregular growth and rest of the isolates showed sparse and regular type except CHBR isolate which showed sparse and irregular type of mycelial growth (Table 3). Basha and Chatterjee (2007) also observed variation in type of mycelial growth as colonies of seventeen isolates were fluffy, whereas three showed compact mycelia. Choudhary and Prasad (2012) also observed two types of mycelial growth as fluffy and compact among different isolates. However, Sharma et al. (2013) observed three types as scattered, smooth and fluffy mycelial growth among different isolates. The results of Kumar et al. (2016) are in agreement with the present study as they have also examined this characteristic in nearly half of the isolates which we have taken afresh from different sites.

In the present study, SGN, RSN, SRS, LDH, NHR, CHBR and RHK isolates showed slow mycelial growth as colony diameter was 12.3, 15.3, 16.3, 17.7, 19.7, 21.2 and 25.0 mm after 48 h of incubation, respectively, while BWN, DBW and HNM isolates showed fast mycelial growth up to 72 h of incubation with colony diameter of 65.0, 62.7 and 62.0 mm after 48 h and colony diameter of 90.0, 79.0 and 86.7 mm after 72 h of incubation, respectively. Mycelial growth and initiation of sclerotia were the fastest in BWN isolate (Table 3). However, all other isolates covered full mycelial growth in the 90 mm diameter Petri plates after 96 h of incubation. Similar trend was also reported by Garg et al. (2010), where they reported significant differences between isolates in relation to the colony diameter measured after 24 and 48 h of incubation. Ahmadi et al. (2012) examined seven populations of *S. sclerotiorum* associated with stem rot of important crops and weeds and based on mycelial growth, these seven populations were classified into four groups i.e. very fast, fast, intermediate and slow growing. Hence, a sufficient variability for cultural characteristics exists not only in the isolates form different hosts but also within the Indian mustard.

**Fig. 1:**
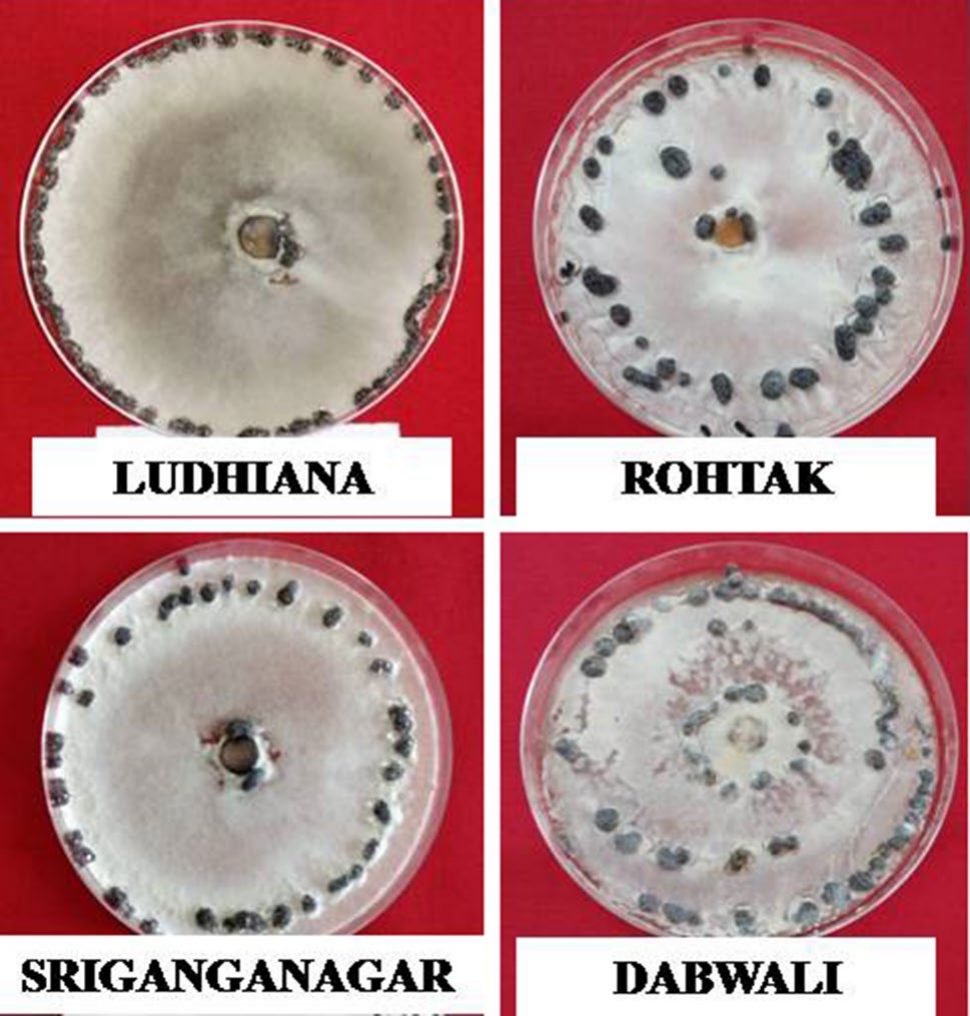
Morphological variability shown by *S. sclerotiorum* isolates. Ludhiana isolate showing—sclerotia attached to rim, Sriganganagar isolate showing—sclerotia near to rim, Dabwali isolate showing—sclerotia in double ring near to rim and centre, Rohtak isolate showing sclerotia scattered all around

The pattern of sclerotia formation varied among the isolates as SGN and HSR isolates formed sclerotia near to rim of Petri plates, while BWL, BWN, FTB, MHG, SRS, LDH, CHBR, HNM and RSN isolates showed sclerotial pattern attached to the rim. RHK and NHR isolates formed sclerotia scattered all around, while DBW isolate formed sclerotia that were double ring, near to rim and centre in Petri plates (Fig. 1). Ghasolia and Shivpuri (2007) also observed variability among 38 isolates of *S. sclerotiorum* collected from Rajasthan, which showed variation in their morphological traits like sclerotial number, size, position and pattern. The differences in morphological aspects among isolates or populations are attributed to variations in fungal genetic, environmental conditions and presence of mycoviruses (Saharan and Mehta 2008). Kumar et al. (2016) also examined sufficient diversity in size of sclerotia and pattern of sclerotia among isolates collected from Indian mustard. In spite of necrotropic nature, wide host range and undoubtedly no selection pressure for change in pathogen population, it appears that isolates have sufficient variability for cultural and morphological characteristics infecting Indian mustard.

### Molecular variability based on Universal Rice Primers (URPs)

**Fig. 2:**
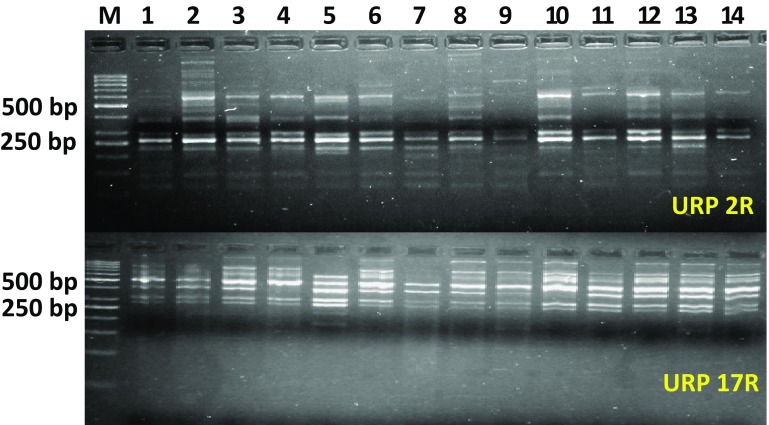
DNA amplification profile of fourteen *S. sclerotiorum* isolates with URP 2R and URP 17R primers. M—50 bp ladder, lane 1 to 14—isolates; 1-CHBR, 2-HNM, 3-MHG, 4-SGN, 5-NHR, 6-BWL, 7-HSR, 8-RHK, 9-FTB, 10-BWN, 11-SRS, 12-LDH, 13-DBW, 14-RSN

The principle of URP-PCR technique is similar to random amplified polymorphic DNA (RAPD) but URP-PCR method has more reproducibility over the RAPD. In the present study, a set of 12 URP primers were used for DNA polymorphism analysis of the fourteen *S. sclerotiorum* isolates. Out of the twelve URP primers, two primers did not give satisfactory amplification. Only ten primers resulted in amplification of distinct and reproducible bands in the present investigation. The primers generated the fragments, which ranged from 150 to 800 bp among *S. sclerotiorum* isolates. Ten primers amplified a total of 57 unambiguous and reproducible bands out of which 49 were polymorphic as indicated in Table 4. The DNA amplification profile of fourteen isolates with primers URP 2R and URP 17R is shown in Fig. 2. The total number of bands observed for each primer ranged from 4 (URP 1 F) to 7 (URP 2R and URP 6R) with an average of 4.75 bands per primer. However, Sharma et al. (2013) reported 385 polymorphic bands out of total 692 scorable amplicons among 17 *S. sclerotiorum* isolates with 50 decamer primers. They observed 13–14 bands/primer with band size range of 180–3900 bp. The reason for large number of bands/primer might be due to less specificity of RAPD primers in comparison to URPs.

**Table 4:** Amplification profile of fourteen *Sclerotinia sclerotiorum* isolates with Universal Rice Primers (URPs)

Sr. no.	Primer	No. of bands/amplicons detected	Polymorphic bands	Monomorphic bands	Percentage polymorphism (%)	Band size range (bp)	PIC value
1	URP 1F	4	3	1	75	250–450	0.716
2	URP 2F	6	6	0	100	180–450	0.815
3	URP 2R	7	5	2	71.4	225–600	0.843
4	URP 4R	5	5	0	100	225–600	0.780
5	URP 6R	7	7	0	100	180–600	0.854
6	URP 9F	5	3	2	60	150–800	0.723
7	URP 17R	6	3	3	50	280–800	0.832
8	URP 25F	6	6	0	100	200–700	0.831
9	URP 30F	5	5	0	100	200–500	0.795
10	URP 38F	6	6	0	100	200–700	0.823

The per cent polymorphic bands among 14 isolates of *S. sclerotiorum* ranged from 50 to 100% similarly, Karimi et al. (2011) reported 59.5–75.21% polymorphic bands in the genetic diversity study of *S. sclerotiorum* populations using rep-PCR genomic fingerprinting. Hence in the present study ten primers selected were very appropriate and highly informative giving a high level of polymorphism. Polymorphic Information Content (PIC) value for the primers varied from 0.716 to 0.854. Colagar et al. (2010) also conducted diversity studies using 18 random primers among 12 *S. sclerotiorum* isolates. Out of them three random primers (Ar0R2, Ar081 and Ar173) contained high polymorphism.

**Fig. 3:**
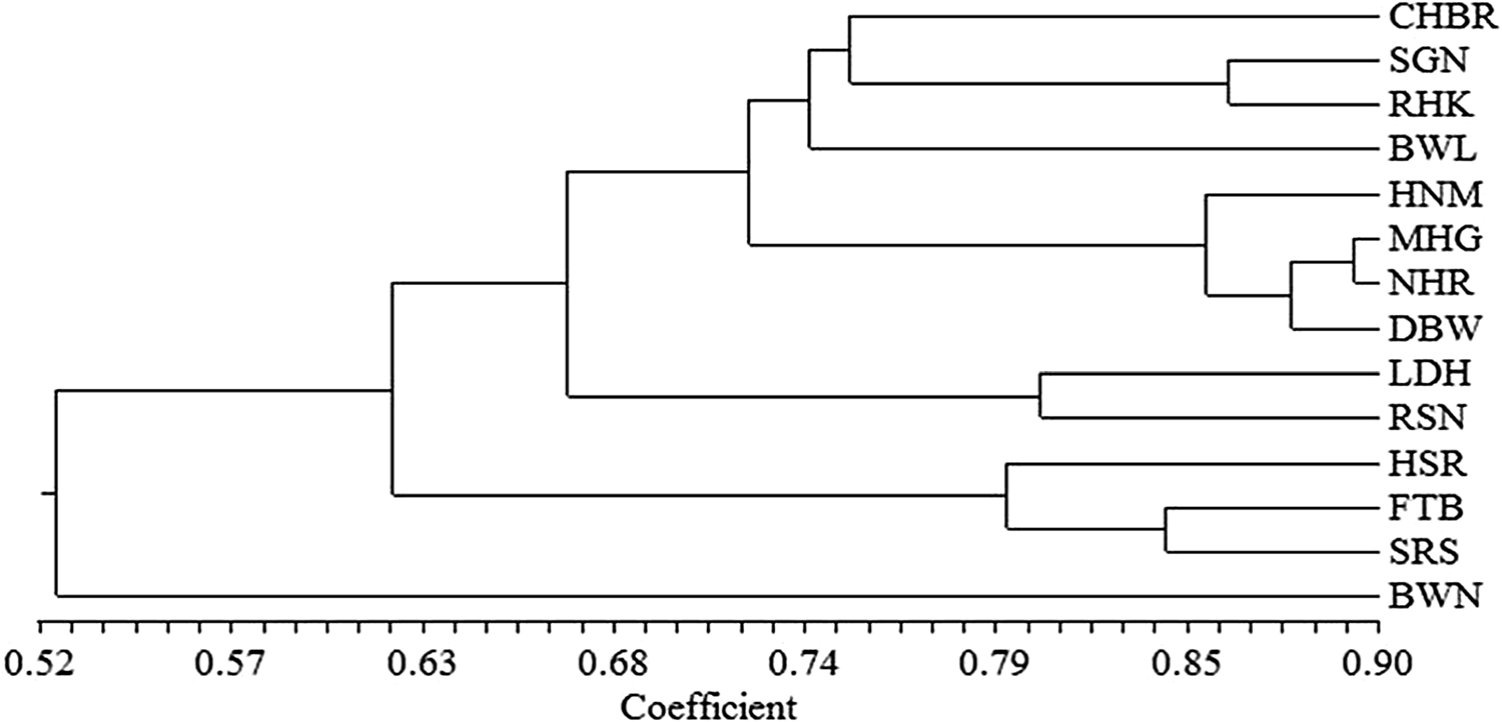
UPGMA dendrogram showing genetic relationship among fourteen *Sclerotinia sclerotiorum* isolates based on Jaccard’s similarity matrix data using ten Universal Rice Primers. The bottom scale is the percentage of similarity (*BWL* Bawal, *BWN* Bhiwani, *DBW* Dabwali, *FTB* Fatehabad, *HSR* Hisar, *MHG* Mahendragarh, *RHK* Rohtak, *SRS* Sirsa, *LDH* Ludhiana, *CHBR* Chhanibari, *HNM* Hanumangarh, *NHR* Nohar, *RSN* Raisingh Nagar, *SGN* Sriganganagar)

Jaccard’s similarity coefficient calculated from the URP data matrix showed that the genetic relatedness among the isolates ranged from 0.41 to 0.89 with an average value of 0.65. Therefore, the genetic diversity observed among the isolates was 35%. At similarity value of 0.52, BWN isolate was out grouped from rest of the thirteen isolates (Fig. 3). The rest of the thirteen isolates were grouped into two clusters at a similarity value of 0.61. Cluster I was further grouped into two sub clusters. Sub cluster I was comprised of following eight isolates CHBR, SGN, RHK, BWL, HNM, MHG, NHR and DBW. Sub cluster II was having only two isolates i.e. LDH and RSN. Cluster II was composed of three isolates i.e. HSR, FTB and SRS. Maximum similarity (0.892) was found between MHG and NHR isolates and minimum similarity was found in between CHBR and BWN (0.410) isolates. Similarly, Litholdo Júnior et al. (2011) studied the genetic variability in 40 *S. sclerotiorum* isolates using 16 RAPD markers. The UPGMA cluster analysis using Jaccard’s genetic distance resulted in separation of the isolates into three clusters. The grouping of isolates depends upon the genetic similarity among the isolates.

### Correlation between cultural, morphological and molecular variability

Cultural, morphological and URPs based molecular diversity revealed that five out of total eight isolates in sub cluster I (HNM, MHG, SGN, BWL and RHK) had similar colony colour i.e. whitish colour. Six isolates in this sub group showed regular growth whether sparse or fluffy while two isolates (CHBR and NHR) showed irregular type of growth. No significant similarity was observed with regard to mycelial growth in this sub cluster, as it varied from 41.3 to 86.7 mm after 72 h of incubation. Variations among the isolates in relation to morphological characters of sclerotia were also observed in this sub cluster. However, Sharma et al. (2013) in RAPD based diversity study with *Sclerotinia* isolates of oilseed *Brassica* found that the very fast growing group of isolates was also within the same genetic cluster except SR-16. Slight deviations from the previous results might be due to the difference in isolates and the difference in markers (URPs) which are longer and more authentic than RAPD markers.

Sub cluster II was having only two isolates i.e. LDH and RSN which showed significant similarity with regard to cultural and morphological traits of the fungus, as these two isolates showed sparse and regular type of growth and moderate spreading of mycelial growth ranging between 40 and 50 mm diameter in culture media at 72 h of inoculation and similarity with regard to sclerotia initiation, their number and pattern in culture.

HSR, SRS and FTB isolates showed similarity in all the cultural and morphological traits as also evident form UPGMA dendrogram. BWN isolate was found to be the most diverse on the basis of morphological, cultural and molecular marker based analysis. Mycelial growth and sclerotia initiation were faster in this isolate. Also, it was out grouped in the dendrogram based on molecular marker analysis. CHBR and BWH isolates had the maximum diversity in most of the cultural and morphological traits which was also indicated by the similarity coefficient value and UPGMA dendrogram.

For the last few years, this disease was considered to be more of myceliogenic nature in all main mustard growing regions of Haryana but recently, the infection on upper parts through ascospores has become more common suggesting variability through hybridization. Hence morphological and cultural variability observed in this study in general was strongly correlated to molecular marker based variability and high levels of genotypic variability might be due to exposure of the pathogen to diverse environments and a wide host range. Therefore, the information harnessed about the pathogen is quite helpful for further utilization in the breeding programs for Sclerotinia stem rot resistance in Indian mustard.

## References

[CR1] Aggarwal R, Sharma V, Kharbikar LL, Renu (2008). Molecular characterization of *Chaetomium* species using URP-PCR. Genet Mol Biol.

[CR2] Agrios GN (2005). Plant pathology.

[CR3] Ahmadi MR, Nik-khah MJ, Aghajani MA, Ghobakhloo M (2012). Morphological variability among *Sclerotinia sclerotiorum* populations associated with stem rot of important crops and weeds. World Appl Sci J.

[CR4] Aldrich-Wolfe L, Travers S, Nelson BD (2015). Genetic variation of *Sclerotinia sclerotiorum* from multiple crops in the North Central United States. PLoS One.

[CR5] Basha SA, Chatterjee SC (2007). Variation in cultural, mycelial compatability and virulence among the isolate of *Sclerotinia sclerotiorum*. Indian Phytopathol.

[CR6] Choudhary V, Prasad L (2012). Morpho-pathological, genetic variations and population structure of *Sclerotinia sclerotiorum*. Int J Plant Res.

[CR7] Colagar AH, Saadati M, Zarea M, Talei SA (2010). Genetic variation of the Iranian *Sclerotinia sclerotiorum* isolates by standardizing DNA polymorphic fragments. Biotechnology.

[CR8] Cubeta MA, Cody BR, Kohli Y, Kohn LM (1997). Clonality in *Sclerotinia sclerotiorum* on infected cabbage in eastern North Carolina. Phytopathology.

[CR9] Garg H, Kohn LM, Andrew M, Li H, Sivasithamparam K, Barbetti MJ (2010). Pathogenicity of morphologically different isolates of *Sclerotinia sclerotiorum* with *Brassica napus* and *B. juncea* genotypes. Eur J Plant Pathol.

[CR10] Ghasolia RP, Shivpuri A (2007). Morphological and pathogenic variability in rapeseed and mustard isolates of *Sclerotinia sclerotiorum*. Indian Phytopathol.

[CR11] Ghasolia RP, Shivpuri A, Bhargava AK (2004). *Sclerotinia* rot of Indian mustard (*Brassica juncea*) in Rajasthan. Indian Phytopathol.

[CR12] Goswami K, Tewari AK, Awasthi RP (2008). Cultural, morphological and pathological variability in isolates of *Sclerotinia sclerotiorum* (Lib.) de Bary. J Mycol Plant Pathol.

[CR13] Jaccard P (1908). Nouvelles recherches sur la distribution florale. Bull Soc Vaudoise Sci Nat.

[CR14] Kang IS, Chahal SS (2000). Prevalence and incidence of white rot of mustard incited by *Sclerotinia sclerotiorum* in Punjab. Plant Dis.

[CR15] Kapatia A, Gupta T, Sharma M, Khan A, Kulshrestha S (2016). Isolation and analysis of genetic diversity amongst *Sclerotinia sclerotiorum* isolates infecting cauliflower and pea. Indian J Biotechnol.

[CR16] Karimi E, Safaie N, Shams-Bakhsh M (2011). Assessment of genetic diversity among *Sclerotinia sclerotiorum* populations in canola fields by rep-PCR. Trakia J Sci.

[CR17] Krishnia SK, Meena PD, Chattopadhyay C (2000). Seed-yield and yield-attributes of Indian mustard affected by *Sclerotinia* rot. J Mycol Plant Pathol.

[CR18] Kumar P, Rathi AS, Singh JK, Berwal MK, Kumar M, Kumar A, Singh D (2016). Cultural, morphological and Pathogenic diversity analysis of *Sclerotinia sclerotiorum* causing Sclerotinia rot Indian mustard. Indian J Ecol.

[CR19] Li Z, Zhang M, Wang Y, Li R, Fernando WGD (2008). Mycelial compatibility group and pathogenicity variation *Sclerotinia sclerotiorum* populations in sunflower from China, Canada and England. J Plant Pathol.

[CR20] Li CX, Liu SY, Sivasithamparam K, Barbetti MJ (2009). New sources of resistance to *Sclerotinia* stem rot caused by *Sclerotinia sclerotiorum* in Chinese and Australian *Brassica napus* and *B. juncea* germplasm screened under Western Australian conditions. Australas Plant Pathol.

[CR21] Litholdo Júnior CG, Gomes EV, Lobo Júnior M, Nasser LCB, Petrofeza S (2011). Genetic diversity and mycelial compatibility groups of the plant-pathogenic fungus *Sclerotinia sclerotiorum* in Brazil. Genet Mol Res.

[CR22] Lodha BC, Bhatanagar MK, Mathur K, Doshi A, Mathur S, Bairwa LN, Sharma D, Trivedi A (1992) Plant pathological thoughts and news. no. 6(3) Department of Plant Pathology, Rajasthan College of Agriculture, Udaipur (India)

[CR23] Meinhardt LW, Wulff NA, Bellato CM, Tsai SM (2002). Telomere and microsatellite primers reveal diversity among *Sclerotinia sclerotiorum* isolates from Brazil. Fitopatol Bras.

[CR24] Morrall RAA, Duczek LT, Sheard JW (1972). Variations and correlations within and between morphology, pathogenicity and pectolytic enzyme activity in *Sclerotinia* from Saskatchewan. Can J Bot.

[CR25] Murray MG, Thompson WF (1980). Rapid isolation of high molecular weight plant DNA. Nucleic Acids Res.

[CR26] Saharan GS, Mehta N (2008). Sclerotinia diseases of crop plants: biology, ecology and disease management.

[CR27] Sambrook J, Fritsch EF, Miniatis T (1989). Molecular cloning a laboratory manual.

[CR28] Sharma P, Meena PD, Kumar S, Chauhan JS (2013). Genetic diversity and morphological variability of *Sclerotinia sclerotiorum* isolates of oilseed *Brassica* in India. Afr J Microbiol Res.

[CR29] Sharma P, Meena PD, Verma PR, Saharan GS, Mehta N, Singh D, Kumar A (2015). *Sclerotinia sclerotiorum* (Lib) de Bary causing sclerotinia rot in Brassicas: a review. J Oilseed Brassica.

[CR30] Shivpuri A, Sharma KB, Chhipa HP (2000). Some studies on the stem rot (*Sclerotinia sclerotiorum*) disease of rapeseed/mustard in Rajasthan. J Mycol Plant Pathol.

[CR31] Singh R, Singh D, Barbetti MJ, Singh H, Caixia L, Sivasithamparam K, Salisbury P, Wayne Burton, Tingdong F (2008). *Sclerotinia* rot tolerance in oilseed *Brassica*. J Oilseeds Res.

[CR32] Thilagavathi R, Nakkeeran S, Raguchander T, Samiyappan R (2013). Morphological and genomic variability among *Sclerotium rolfsii* populations. Bioscan.

[CR33] Willetts HJ, Wong JAL (1980). The biology of *Sclerotinia sclerotiorum*, *S. trifoliorum* and *S. minor* with emphasis on specific nomenclature. Bot Rev.

[CR34] Yli-Mattila T, Hannukkala A, Paavanen-Huhtala S, Hakala K (2010). Prevalence, species composition, genetic variation and pathogenicity of clover rot (*Sclerotinia trifolioum*) and *Fusarium* spp. in red colver in Finland. Eur J Plant Pathol.

[CR35] Ziman L, Jedryezka M, Srobarova A (1998). Relationship between morphological and biochemical characterstics of *Sclerotinia sclerotiorum* isolates and their aggressivity. Z Pfla Krank Pfa Schutz.

